# The Effect of Pyrite Content in Aggregates on Concrete Deformation and Failure Prediction

**DOI:** 10.3390/ma19101969

**Published:** 2026-05-10

**Authors:** Kai Zhang, Wei Li, Shaoping Wang, Conglin Wang, Xiaojun Huang, Min Zhu, Zhixin Wang, Min Deng

**Affiliations:** 1 College of Materials Science and Engineering, Nanjing Tech University, Nanjing 211816, China; 2 Nanshan Mining Company Ltd., Anhui Maanshan Iron and Steel Mining Resources Group, Maanshan 243000, China

**Keywords:** pyrite, aggregates, concrete, service life prediction, the activation energy

## Abstract

Iron ore mining requires the surrounding rock to be excavated, and the beneficiation process generates tailings. When used as construction aggregates, these materials can cause concrete to crack due to the presence of pyrite. Currently, there are no established technical methods to prevent damage caused by pyrite, which limits the resource recovery of such solid waste. In this study, we selected the surrounding rock and tailings to serve as coarse or fine aggregates for C50 concrete based on standard engineering mix proportions. We found that surface-exposed pyrite on aggregates oxidizes first to form ettringite, triggering expansion, with the expansion rate positively correlated with the surface-exposed pyrite content. The deformation process was quantitatively characterized using the Arrhenius equation and by analyzing the acceleration effect of temperature on expansion, yielding an apparent activation energy of 8.28–9.47 kJ/mol. Using a 0.04% expansion value as the failure criterion, the results indicate that at an annual average temperature of 20 °C, C50 concrete with surface-exposed pyrite introduced by concrete aggregates exceeding 20 kg/m^3^ will fail within its service life.

## 1. Introduction

As a representative iron sulfide mineral, pyrite is one of the most widely distributed minerals in the Earth’s crust and is commonly found in association with iron ore or natural rock. A certain amount of pyrite often remains in the surrounding rock and tailings after the mining and beneficiation of iron ore. The surrounding rock and tailings have some remarkable properties, such as high strength, high elastic modulus, and good wear resistance, making them suitable for use as concrete aggregates, generally referred to as iron sulfide-containing aggregates. However, under the influence of moisture and oxygen, these aggregates can trigger iron sulfide oxidation and sulfate corrosion within the concrete. This leads to potential risks, including concrete expansion, ultimately compromising the durability of concrete structures. Pyrite—a harmful component—is often ignored during the utilization of these aggregates, which are widely used in civil construction and even in high-strength concrete with specific requirements, such as bridges and roads. As the service life of buildings extends, concrete damage becomes increasingly apparent. There are frequent cases of concrete deterioration caused by pyrite oxidation around the world.

In Quebec, Canada, iron sulfide oxidation has caused damage to numerous residential buildings, affecting more than 20,000 families [1,2,3,4]. In eastern Connecticut, USA, the foundation of over 400 houses deteriorated due to this oxidation [5,6], with repair costs reaching hundreds of thousands of dollars per household. In Switzerland, a dam constructed with sulfur-containing aggregates experienced continuous expansion since its completion [7]. On a highway bridge in Gloucestershire, UK, deterioration of the foundation concrete due to pyrite oxidation was discovered [8], with the affected depth reaching 50 mm. In Romania, asphalt pavements prepared with materials containing pyrite developed noticeable yellow pop-outs after prolonged exposure to rainwater [9]. BS EN 12620: 2013 [10] specifies that if pyrite is present in the aggregate, the total sulfur content of the aggregate shall not exceed 0.1%.

Previous studies have generally confirmed that pyrite oxidation occurs through reactions with water and oxygen in the environment, resulting in the formation of Fe^2+^, SO_4_^2−^, and H^+^, as shown in Equation (1) [11,12].

2FeS_2_ + 7O_2_ + 2H_2_O → 2Fe^2+^ + 4H^+^ + 4SO_4_^2−^ (1)

The Fe^2+^ produced is unstable in the environment and rapidly oxidizes to form Fe^3+^ in the environment, as shown in Equation (2).

4Fe^2+^ + O_2_ + 4H^+^ → 4Fe^3+^ + 2H_2_O (2)

As Equation (1) produces a large amount of H^+^, the acidity of the environment gradually increases. When the pH falls below 3.5, Fe^3+^ acts as a strong oxidizing agent to further oxidize pyrite, as shown in Equation (3).

FeS_2_ + 14Fe^3+^ + 8H_2_O → 15Fe^2+^+ 16H^+^+ 2SO_4_^2−^ (3)

As sulfate ions continue to form, they react with the calcite produced by silicate carbonation to form gypsum, which then reacts with the aluminates in the concrete to form calcium aluminate hydrate, as shown in Equations (4) and (5). The molar volume of calcium aluminate hydrate is 3–8 times that of the initial solid reactants, which can easily lead to concrete expansion and failure [12,13,14].

2X + SO_4_^2−^ + CaCO_3_ + 2H_2_O → CaSO_4_·2H_2_O + CO_3_^2−^ + 2X (4)

3CaSO_4_·2H_2_O + (CaO)_3_(Al_2_O_3_) + 26H_2_O → 3CaO·Al_2_O_3_·3CaSO_4_·32H_2_O (5)

where X = Na^+^, K^+^, or 1/2 Mg^2+^.

To ensure safety in aggregate applications, research into whether aggregates containing pyrite can be used in construction and how to prevent concrete deterioration caused by pyrite oxidation continues to advance [15,16,17,18]. However, most current methods typically involve rapid oxidation using aggregates within a limited particle size range. In engineering practice, concrete aggregates consist of a full mix of sizes, which differs from the particle size ranges selected for laboratory experiments. Some methods use chemical agents such as strong oxidizing agents to accelerate oxidation, which fails to simulate the actual conditions encountered in concrete applications. Consequently, it is difficult to predict the behavior of pyrite in aggregates.

Determining pyrite content is particularly important for quickly assessing whether it poses a risk. With the aid of advanced instruments, it is possible to quantify its content in aggregates, but rapid measurement and implementation are not feasible in practical engineering applications [19,20]. Recently, Wang et al. [21] proposed a method for determining pyrite content without using advanced instruments, providing a practical experimental method for defining the safety threshold of pyrite content.

The failure prediction model for concrete is one of the most effective methods for determining the suitability of aggregates. In 1889, Swedish chemist Arrhenius proposed the Arrhenius equation, which describes the exponential relationship between the reaction rate constant and temperature, establishing the physical phenomenon that an increase in temperature effectively accelerates the reaction rate. Currently, the influence of temperature on the expansion kinetics of ASR (alkali–silicate reaction) can be expressed using this equation, a finding that has been verified by numerous scholars [22,23]. This expansion model is widely applied in predicting the damage caused by ASR reactions.

In this study, we prepared concrete specimens using coarse or fine aggregates derived from the surrounding rock or tailings with different pyrite contents. By controlling the curing temperature to accelerate pyrite oxidation, we investigated the oxidation mechanism based on concrete deformation data and SEM-EDS microscopic results. By analyzing the expansion results of concrete at different temperatures, we established a failure prediction model for concrete based on the Arrhenius equation, and derived a safety threshold for using aggregates containing pyrite.

## 2. Materials and Methods

### 2.1. Materials

#### 2.1.1. Aggregates

The aggregates were sourced from the surrounding rock and tailings generated from the mining and beneficiation processes at two open-pit iron ore mines located in the same ore vein in Anhui, China. The size of the surrounding rock and tailings is 50–200 mm and 0–28 mm, respectively. Following the steps of crushing (PE60*100, Wuxi Jianyi Instrument & Machinery Co., Ltd., Wuxi, China), screening (ISO-679, Wuxi Jianyi Instrument & Machinery Co., Ltd., Wuxi, China), blending, and mixing based on the sample particle size, 5–25 mm continuously graded crushed rock were prepared as coarse aggregates, and sand with a fineness modulus of 2.80 was prepared as fine aggregates in concrete. To ensure the uniform distribution of pyrite in the aggregates, both aggregates were mixed separately using the quartering method. To distinguish the varying oxidative effects caused by pyrite in aggregates of different particle sizes, pyrite-containing coarse and fine aggregates were used separately. When the coarse aggregates are prepared from the surrounding rock or tailings, the fine aggregates used should be pyrite-free natural river sand. When the surrounding rock or tailings are used as fine aggregates, the coarse aggregates should be prepared with pyrite-free limestone. The configuration of aggregates in the concrete is shown in Table 1 and Table 2.

**Figure 1:**
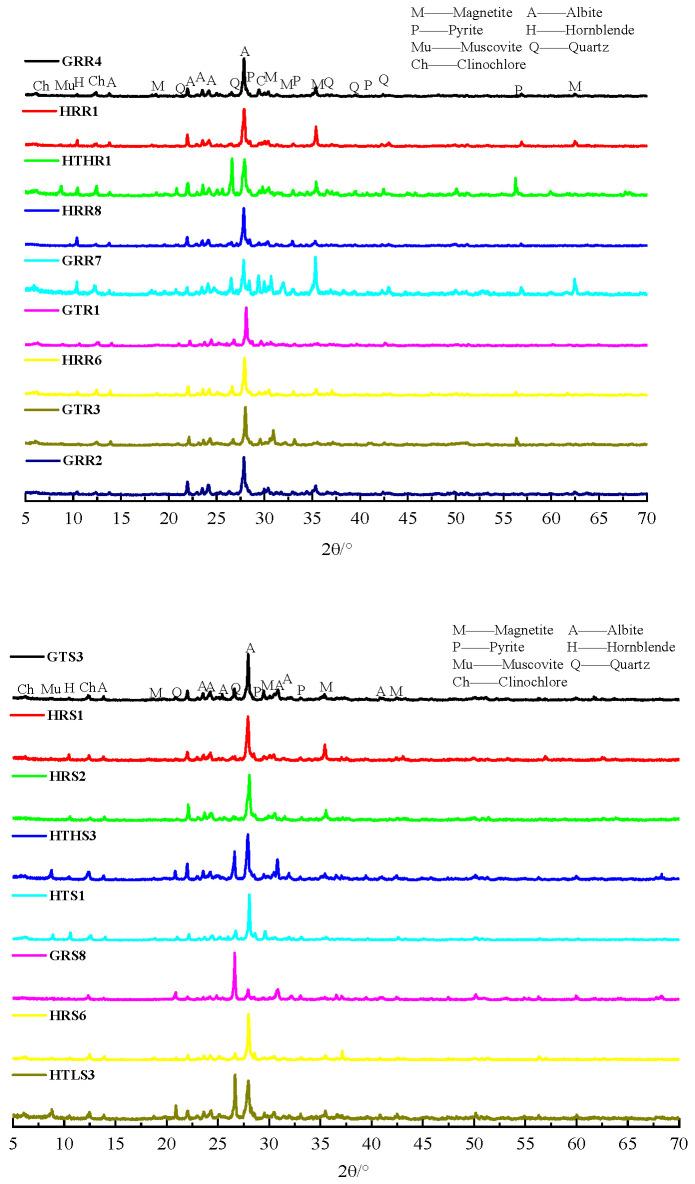
The XRD patterns of aggregates prepared from the surrounding rock or tailings.

The aggregates were coded by origin and type: the initial letters G and H distinguish the two mines sampled, while the final letters R and S denote coarse aggregates (crushed rock) and fine aggregates (manufactured sand), respectively. For example, GRR4 represents 5–25 mm continuously graded crushed rock, which was used as coarse aggregates sourced from the G mine. The mineralogical compositions of the aggregates were analyzed by X-ray diffraction (XRD; DX-2700BH, Dandong Haoyuan Instrument Co., Ltd., Dandong, China). The XRD patterns of the aggregates are shown in Figure 1, which show that they consist primarily of feldspar and contain small amounts of hornblende, chlorite, mica, calcite, quartz, magnetite, and pyrite.

**Figure 2:**
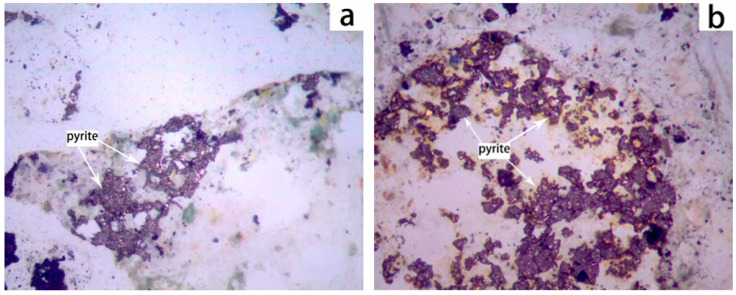
The distribution of pyrite in aggregates prepared from the surrounding rock or tailings. The pyrite in (**a**) coarse aggregates and (**b**) fine aggregates.

The aggregates were bonded with epoxy resin, and following the hardening of the resin, the samples were sectioned and polished into thin sections for observation (AutoMet 250, Buehler, Lake Bluff, IL, USA) under a polarizing microscope (DM750P, Leica Microsystems, Wetzlar, Germany). Figure 2a,b illustrates the distribution of pyrite within the aggregates. Pyrite is distributed throughout the aggregates in a dispersed manner; some areas exhibit a mosaic structure and some pyrite particles are in a free state, while others are partially exposed on the surfaces of aggregates prepared from the surrounding rock and tailings.

#### 2.1.2. Cement and Admixtures

**Figure 3:**
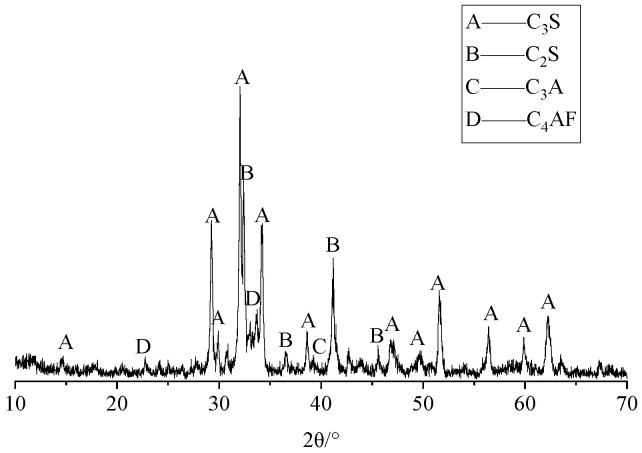
The XRD pattern of the cement.

We selected P·I 42.5 standard cement from Fushun Cement Co., Ltd. (Fushun, China), with a tricalcium aluminate (C_3_A) content of 6.8%. Figure 3 shows its XRD results, with a composition of mainly C_3_S, C_2_S, C_3_A, and C_4_AF. The admixtures used were Grade II fly ash and S95 slag powder, supplied by Nanjing Chemical Industry Park Thermal Power Generation Co., Ltd. (Nanjing, China) and Nanjing Meibao New Building Materials Co., Ltd. (Nanjing, China), respectively. Table 3 lists the chemical compositions of the cement and admixtures. The superplasticizer used in the test was a polycarboxylate admixture from Jiangsu Sobote New Materials Co., Ltd. (Nanjing, China), with a water-reduction rate of 25%.

### 2.2. Experimental Methods

#### 2.2.1. Determining the Pyrite Content in Aggregates

The aggregates prepared from the surrounding rock or tailings were immersed in a 1:1 HNO_3_ solution at 80 °C for 1 h, where the pyrite was essentially completely dissolved after immersion. The solution then reacted with an excess of saturated BaCl_2_ solution to ensure complete precipitation. The pyrite content in the aggregates was determined gravimetrically based on the mass of the BaSO_4_ precipitate obtained after filtration, washing, and calcination at 950 °C. Considering the diverse occurrences of pyrite within the aggregates, two testing methods were employed. The total pyrite content was measured after grinding the graded samples to pass a 0.075 mm sieve. Conversely, the surface-exposed pyrite content was determined by testing the graded samples directly.

#### 2.2.2. Concrete Casting and Curing

The concrete specimens (75 mm × 75 mm × 285 mm) were cast according to the mix design referenced from actual engineering proportions, as detailed in Table 4. After molding, the specimens were cured in humid air at 20 ± 2 °C for 48 h before demolding, and then for 14 days, after which their initial length was measured. Over the next 364 days, the length of the specimens was measured at 14, 28, 56, 91, 182, 273, and 364 days of age, and their expansion ratio was calculated using

(6) $$\varepsilon_{t}=\frac{L_{t}-L_{0}}{L}\times 100\%$$

where ε_t_ is the expansion ratio of the test specimen at t_d_ (%); L_t_ represents its length at t_d_ (mm); L_0_ denotes its initial length (mm); and L is its effective length (265 mm).

In this study, specimens made of coarse aggregate GRR2 and natural river sand (fine aggregate) or fine aggregate HTLS3 and coarse aggregate limestone were cured in thermostatic curing chambers (ZKY-400 Steam Rapid Curing Chamber, Hebei Yiqishun Test Instrument Co., Ltd., Cangzhou, China) at 20, 30, 40, 50, 60, and 70 °C, respectively. All other specimens were cured at 70 °C. The relative humidity of the environment was uniformly maintained at 75 ± 5% using a saturated solution of NaCl [24].

#### 2.2.3. Microstructural Analysis

After curing for different durations, samples were taken from the concrete specimens with varying pyrite content, cured at different temperatures. The samples were dried at 105 °C for 12 h to remove moisture, and were then embedded in epoxy resin within a mold and cured in an oven at 40 °C to form stable blocks. After the sample was polished into a thin section, a polarizing microscope was used to analyze the oxidation patterns of pyrite under various factors. The oxidation process of pyrite was analyzed using an ULTRA-55 field emission scanning electron microscope (Carl Zeiss AG, Oberkochen, Germany) equipped with the Oxford X-MaxN EDS detector (Oxford Instruments, Abingdon, UK) at an acceleration voltage of 5–10 kV and a working distance of 8.5–10 mm, focusing on its microstructure, elemental distribution, and crystal structure.

## 3. Results and Discussion

### 3.1. Activation Energy for Pyrite Oxidation

#### 3.1.1. The Effect of Temperature on Pyrite Oxidation

**Figure 4:**
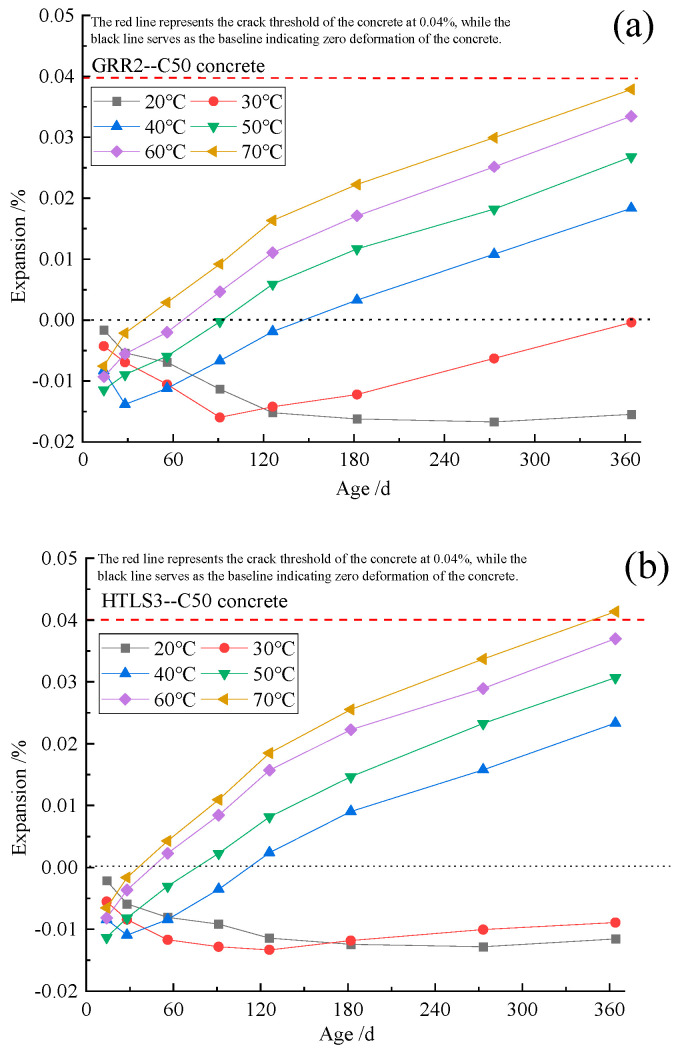
The expansion ratio of (**a**) GRR2 and (**b**) HTLS3 concrete cured at 20–70 °C for 364 d.

**Figure 5:**
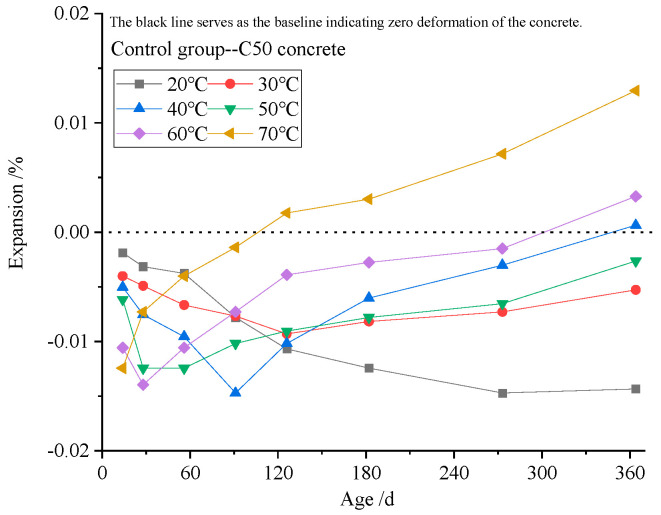
The expansion ratio of the control group concrete cured at 2070 °C for 364 d.

Figure 4 presents the expansion ratio of concrete specimens cured at 20–70 °C for 364 d, prepared from (a) coarse aggregate GRR2 with natural river sand (fine aggregate) and (b) limestone (coarse aggregate) with fine aggregate HTLS3. At 20 °C, the concrete exhibits only shrinkage as the curing age increases, and this shrinkage tends to intensify. At 30–40 °C, the specimens first contract and then exhibit increasing expansion with curing age, with the expansion continuing to increase at 50–70 °C. Higher curing temperatures lead to a greater expansion ratio, indicating that curing temperature has a promoting effect on the expansion of the specimens. The temperature-accelerating effect on the reaction is similar to that observed in ASR failure prediction tests [22,23]. Figure 5 illustrates the expansion of the control group specimens cured at 20–70 °C for 364 d. The expansion of these specimens, which were prepared with coarse aggregate limestone and natural river sand (fine aggregate), increased slightly with curing temperature, but remained minimal overall. 

#### 3.1.2. Concrete Expansion Rate Constant

**Figure 6:**
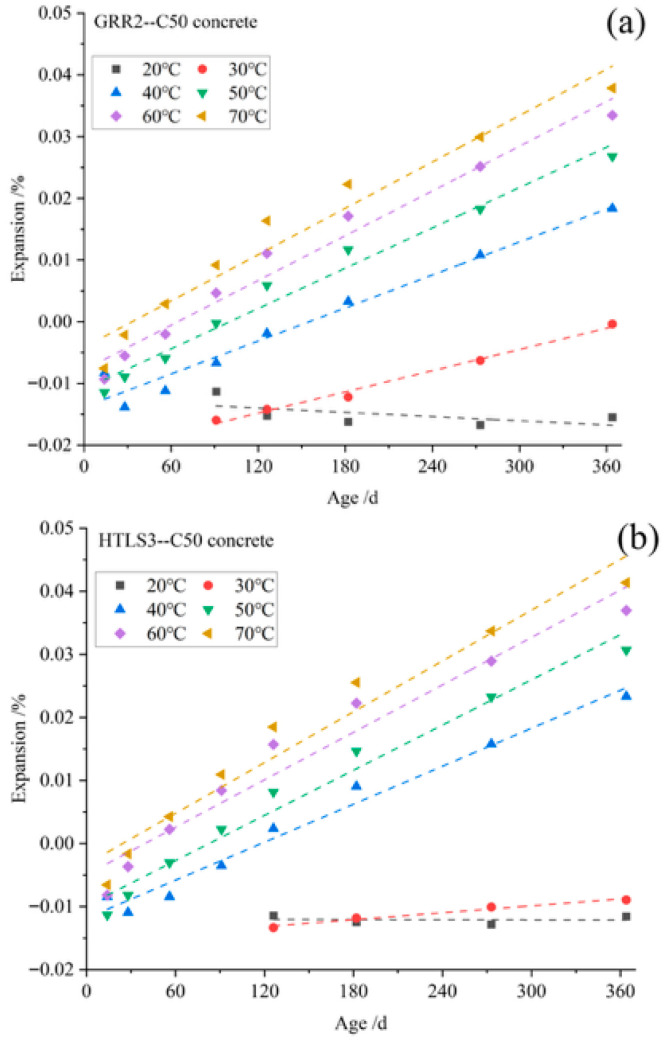
Fitting results of the expansion rate of (**a**) GRR2 and (**b**) HTLS3 concrete cured at 20–70 °C for 364 d.

The expansion rates of concrete, prepared with GRR2 and HTLS3, were linearly fitted for curing durations up to 364 days from 20 to 70 °C. The fitting results are shown in Figure 6 and Table 5. At 20 and 30 °C, the expansion rate remained relatively low, possibly because lower temperatures have a limited effect on promoting pyrite expansion and are offset by the concrete’s own shrinkage. The expansion of some concrete specimens cured at 20 and 30 °C did not even increase with prolonged curing time. However, at curing temperatures of 40–70 °C, the expansion rate exhibited a strong linear correlation with curing time (R^2^ = 0.97429–0.98897). The expansion rate is positively correlated with temperature, confirming the promoting effect of the higher temperature on expansion.

**Figure 7:**
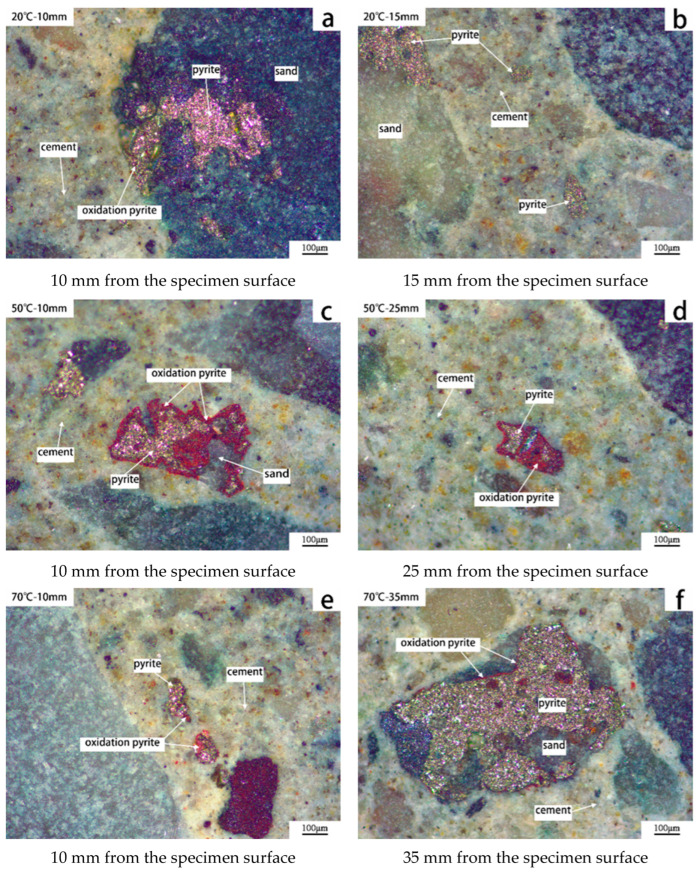
Effect of temperature on pyrite oxidation in concrete prepared with HTLS3 after 364 days of curing. (**a**,**b**) curing at 20 °C, (**c**,**d**) curing at 50 °C, (**e**,**f**) curing at 70 °C.

To verify the accelerating effect of temperature on oxidation, C50 concrete specimens prepared with HTLS3 were cured at three different temperatures (20, 50, and 70 °C). Thin sections from these specimens were subsequently compared to observe pyrite oxidation. Figure 7 illustrates pyrite oxidation in C50 concrete specimens, showing that at 20 °C, pyrite exposed on the sand surface at a depth of 10 mm undergoes significant oxidation, forming red products, whereas no significant oxidation occurs at a depth of 15 mm (Figure 7a,b). At 50 °C, the oxidation depth of surface-exposed pyrite extends to 25 mm, while pyrite that is entirely encapsulated within other minerals in the aggregates or at greater depths remains unoxidized (Figure 7c,d). At 70 °C, the oxidation depth reaches 35 mm, with exposed pyrite showing varying degrees of brown oxidation products (Figure 7e,f). These findings suggest that higher curing temperatures facilitate oxygen migration within the concrete, thereby accelerating the oxidation of exposed pyrite in the sand.

#### 3.1.3. Activation Energy of Swelling

Section 3.1.1 and Section 3.1.2 collectively confirm that elevated temperature accelerates pyrite-induced expansion. This is consistent with the chemical kinetic processes governed by the Arrhenius equation, which defines the relationship between temperature and reaction rate (Equation (7)).

(7) $$k=Ae^{{-E}_{a}/RT}$$

where A represents the pre-exponential factor; R denotes the gas constant (8.314 J·mol^−1^·K^−1^); E_a_ is the activation energy; and T is the absolute temperature of curing. Taking the natural logarithm of both sides of Equation (7) yields

(8) $$\ln k=\ln A-\frac{E_{a}}{R}\times \frac{1}{T}$$

**Figure 8:**
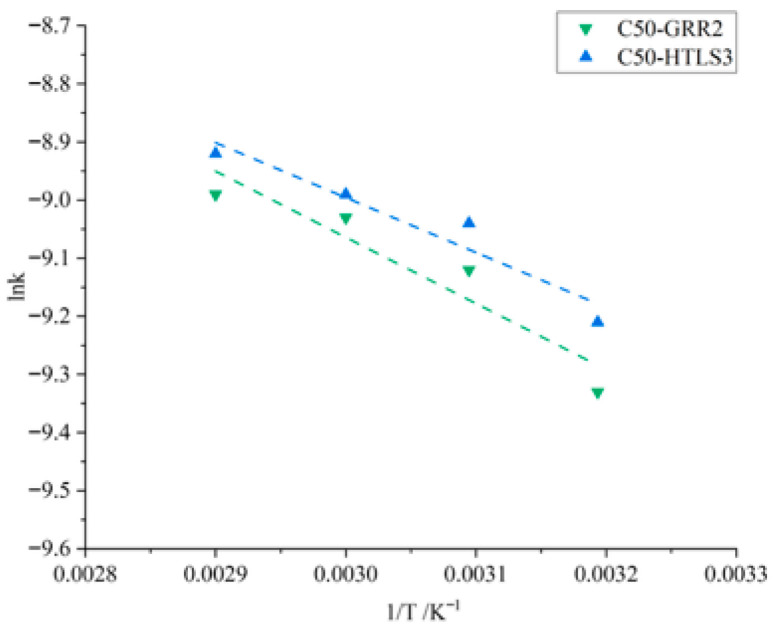
Correlation between ln k and 1/T for concrete prepared with GRR2 and HTLS3.

Equation (8) indicates that a linear relationship can exist between ln k and 1/T. The activation energy E_a_ can be derived from the slope (−E_a_/R). Only the data from the 40–70 °C range were used for Arrhenius fitting as no significant temperature-accelerating effect was observed at 20 and 30 °C. As shown in Figure 8 and Table 6, ln k exhibits a strong linear relationship with 1/T. For concrete prepared with GRR2, the correlation coefficient (R^2^) is 0.94405, yielding an E_a_ of 9.47 kJ/mol. For concrete prepared with HTLS3, (R^2^) reaches 0.96811, with a calculated E_a_ of 8.28 kJ/mol.

### 3.2. Expansion of Concrete with Different Pyrite Contents at 70 °C

To determine whether pyrite that is entirely encapsulated within other minerals in the aggregates causes a risk to concrete when it does not come into contact with oxygen, we quantified both the total pyrite content and the surface-exposed pyrite content of the selected samples, as shown in Table 7.

#### The Influence of Pyrite Content on Concrete

**Figure 9:**
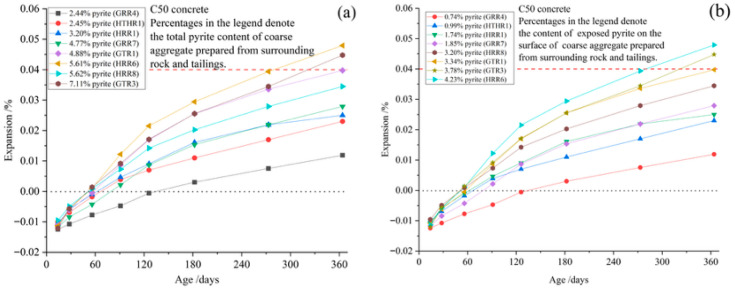
Expansion ratio of concrete prepared with coarse aggregates from surrounding rock or tailings mixed with natural river sands (fine aggregates) cured at 70 °C for 364 d. (**a**) Relationship between concrete expansion ratio and the total pyrite content of coarse aggregate. (**b**) Relationship between concrete expansion ratio and the content of exposed pyrite on the surface of coarse aggregate.

**Figure 10:**
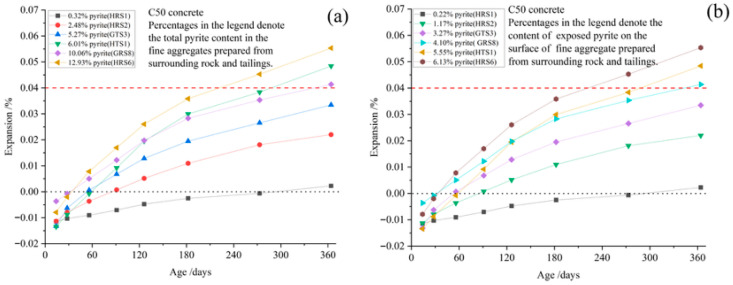
Expansion ratio of concrete prepared with fine aggregates from surrounding rock or tailings mixed with limestones (coarse aggregates) cured at 70 °C for 364 d. (**a**) Relationship between concrete expansion ratio and the total pyrite content of fine aggregate. (**b**) Relationship between concrete expansion ratio and the content of exposed pyrite on the surface of fine aggregate.

Aggregates with varying pyrite contents (Table 7) were used to prepare C50 concrete specimens and subjected to accelerated curing at 70 °C and 75 ± 5% RH for 364 days. Figure 9 and Figure 10 illustrate the resulting expansion rates. The red line represents the crack threshold of the concrete at 0.04%, while the black line serves as the baseline indicating zero deformation of the concrete. Specifically, the expansion rate correlated more strongly with surface-exposed pyrite content than with the total pyrite content. This suggests that surface-exposed pyrite oxidizes preferentially, whereas pyrite that is entirely encapsulated within other minerals in the aggregates may remain inert and harmless to the concrete.

**Figure 11:**
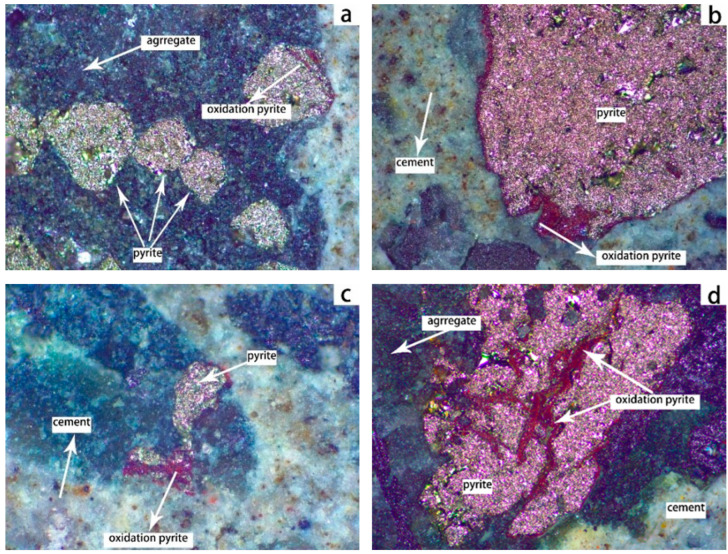
Surface-exposed pyrite-oxidated aggregates after 364 days of curing at 70 °C. (**a**) The pyrite enclosed within the aggregate reacts only at its surface. (**b**,**c**) Reactions occur on the surface of free pyrite. (**d**) The pyrite particles are surrounded by the aggregate and contain cracks connecting to the cement, a process that leads to oxidation.

To verify this hypothesis, we used a polarizing microscope to examine pyrite oxidation within the concrete (Figure 11). The results showed that pyrite underwent oxidation from the surface inward whenever it was located at microcrack sites within the aggregates, exposed on the aggregate surface, or present as free particles. At the same time, pyrite fully enclosed inside the other mineral showed no evident oxidation, indicating that the dense structure effectively prevents pyrite from making contact with water and oxygen. Therefore, the content of surface-exposed pyrite and free pyrite is an important parameter influencing the long-term stability of concrete.

### 3.3. Oxidation Mechanism of Pyrite

**Figure 12:**
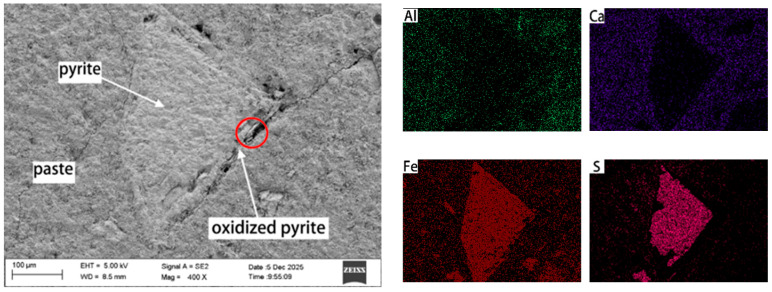
A single pyrite particle from HRS6 and cement paste, and the distribution of elements Al, Ca, Fe, and S in C50 concrete cured in a moist chamber with 75 ± 5% RH at 70 °C for 364 days.

To identify the oxidation products of pyrite and understand the mechanisms behind concrete deterioration, we employed SEM-EDS to analyze the microstructure and elemental distribution of these products. Figure 12 shows the distribution of free pyrite particles, cement paste, and the elements Ca, Al, Fe, and S in C50 concrete (prepared with fine aggregate HRS6 and coarse aggregate limestone) after 364 days of curing at 70 °C and 75 ± 5% RH. A comparison of Fe and S distribution maps shows that Fe in the free pyrite particles underwent no significant migration, whereas S diffused into a 10 μm wide zone surrounding the particles. This indicates that a reaction occurred adjacent to the pyrite particles, with Fe remaining in situ while S migrated out of the reaction zone. No migration of Ca or Al into the pyrite reaction zone was observed.

**Figure 13:**
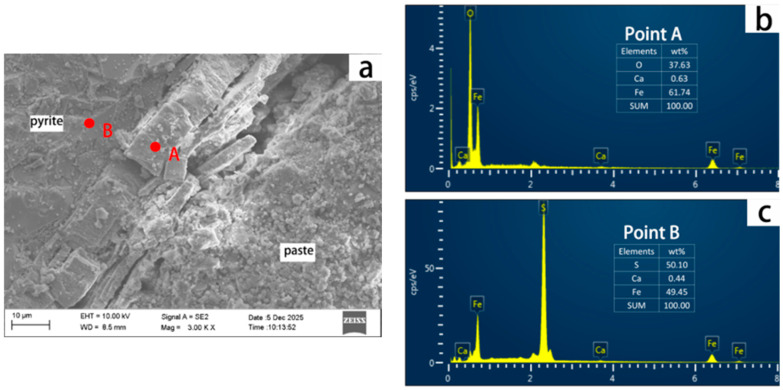
SEM image of a single pyrite particle from HRS6 and cement paste in C50 concrete cured in a moist chamber with 75 ± 5% RH at 70 °C for 364 days (**a**). (**b**,**c**) are the elemental composition of dots A and B.

The top image in Figure 13 is an enlarged view of the area circled in Figure 12. Elemental analysis indicates that the material at point A consists of 61.74% Fe, 37.63% O, and 0.63% Ca, which is close to the composition of goethite (Fe:O:H = 56:32:1), and is a product formed by pyrite oxidation. The material at point B, consisting of 49.45% Fe, 50.10% S, and 0.44% Ca, is consistent with the composition of pyrite with an Fe:S ratio of approximately 7:8, representing unoxidized pyrite. This study confirms that pyrite oxidation produces goethite, which exhibits distinct morphology and elemental signatures. This finding aligns with the results reported by Arezki et al. [13].

**Figure 14:**
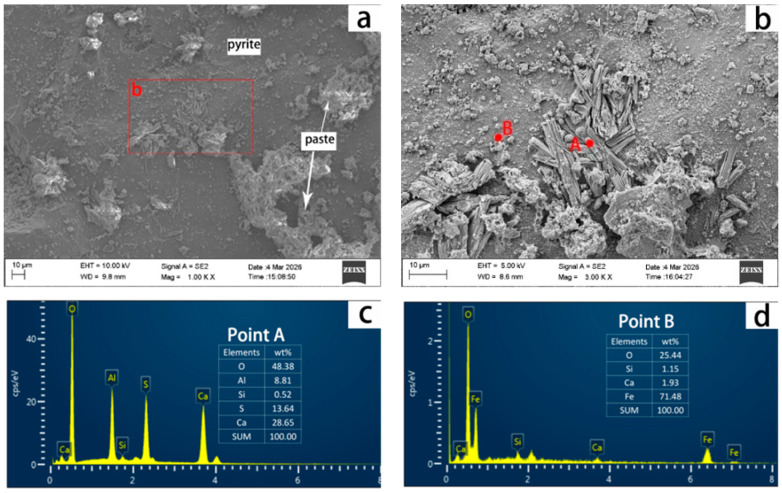
SEM image of the side of pyrite particles at the interface zone between a single pyrite particle and cement pastes in C30 concrete prepared with HRS6 cured in a moist chamber with 75 ± 5% RH at 70 °C for 364 days (**a**). (**b**) is the enlarged view of the selected area in (**a**). (**c**,**d**) are elemental compositions of dots A and B.

Figure 14 shows the microstructure of C30 concrete (prepared with HRS6) after 364 days of curing at 70 °C and 75 ± 5% RH. Image (a) shows a pyrite particle at the interface between the mineral and cement paste, with minor cement paste adhesion. Image (b) is an enlarged view of the circled area in (a). The results indicate the presence of a columnar substance (point A) at the interface between pyrite particles and the cement paste. This substance is composed of 28.65% Ca, 8.81% Al, 13.64% S, 48.38% O, and 0.52% Si, and the Ca:S:Al ratio (14.5:7:4.5) is similar to that of ettringite (17.9:5.3:4.5). Therefore, this columnar substance was presumed to be ettringite. The substance at point B, consisting of 71.48% Fe, 25.44% O, 1.93% Ca, and 1.15% Si, is consistent with the composition of goethite, and is a product formed by pyrite oxidation. This study confirms that ettringite formation, triggered by the release of sulfate ions, is the primary cause of concrete cracking. Characterized by specific morphology and elemental signatures, this result is consistent with the findings of Rodrigues et al. [1,2].

### 3.4. The Failure Prediction Model and Safety Thresholds

CSA A23.2 [25] and ASTM C1293 [26] define a concrete expansion rate of 0.040% or greater as the threshold for damage due to alkali–aggregate reaction. Once this rate is exceeded, cracking typically occurs, leading to a reduction in the structure’s load-bearing capacity. Therefore, 0.040% was adopted as the criterion for concrete failure.

#### 3.4.1. Predictive Models for Damage

The deterioration of concrete due to pyrite oxidation is influenced by pyrite content exposed at the aggregate surface, curing temperature and humidity, curing age, and mix design. For a specific mix design under constant environmental variables, the sulfate erosion of concrete caused by pyrite oxidation is primarily determined by curing temperature (T) and age (t), as expressed by

E = f (T, t)(9)

where E is the expansion ratio, T is the curing temperature, and t is the curing age.

Figure 9 and Figure 10 show that the concrete expansion follows the exponential relationship expressed in

(10) $$E=K(1-e^{-k\left(T\right)t})$$

where K is the characteristic constant and k(T) is the concrete expansion rate constant. Assuming that t1 and t2 are the durations required for concrete to reach the same expansion at temperatures of T1 and T2, respectively,

(11) $$t2=\frac{k(T1)}{k(T2)}t1$$

The expansion rate constant K(T) follows Equation (7), which allows Equation (11) to be rewritten as

(12) $$t2=t1\times e^{\frac{E_{a}(T1-T2)}{RT1T2}}$$

where T2 denotes the annual average temperature (20 °C) at the concrete site, and T1 denotes the accelerated test temperature (70 °C). By determining the time to reach the failure expansion threshold (0.04%) at an accelerated curing temperature (T1) and identifying the activation energy (Table 6), the time to concrete failure under the annual average ambient temperature (T2) can be calculated via Equation (12). This duration was taken as the estimated service life of the concrete.

**Figure 15:**
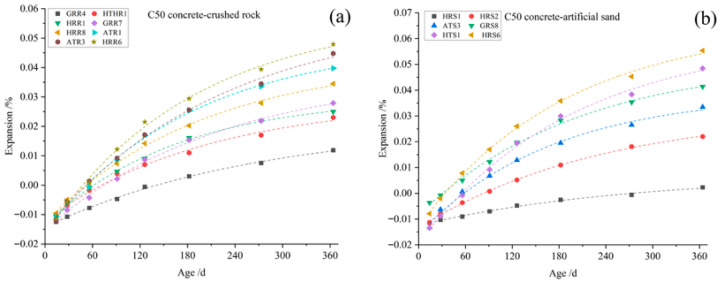
Correlation between expansion rate and curing age of C50 concrete. (**a**) concrete prepared with crushed rock and natural river sand. (**b**) concrete prepared with artificial sand and limestone.

Figure 15 shows the fitting results of concrete expansion rates at different curing ages using a power-law function (y = A × exp(x/t) + y_0_). Based on the fitted function, the specimens whose expansion rates reached the 0.04% threshold are summarized in Table 8. The correlation coefficients (R^2^) ranged from 0.99694 to 0.99848, indicating high fitting accuracy.

#### 3.4.2. Determining the Pyrite Content Threshold

**Figure 16:**
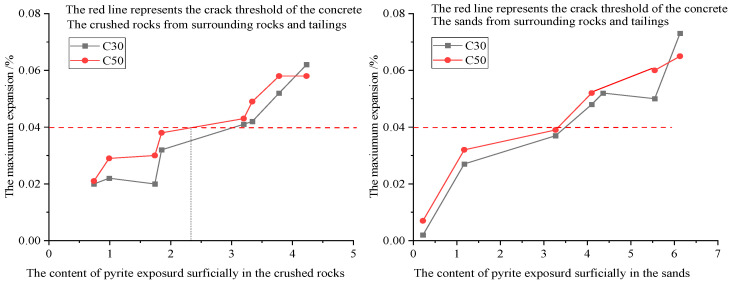
Relationship between maximum concrete expansion and surface-exposed pyrite content in aggregates.

In Table 6, the activation energy for expanding C50 concrete incorporating natural river sand (fine aggregates) and coarse aggregates from the surrounding rock or tailings is 9.47 kJ/mol, while that of concrete using coarse aggregate limestone and fine aggregates from the surrounding rock or tailings is 8.28 kJ/mol. Based on the activation energy results, the service life of concrete at 20 °C can be estimated by substituting the failure time at 70 °C into Equation (12). The results are presented in Table 9. Based on the fitting results in Figure 15, the relationship between the maximum expansion rate (y_0_) of concrete and the surface-exposed pyrite content in the aggregates is established in Figure 16. In environments with an average temperature of 20 °C, concrete incorporating coarse aggregates (containing ≥3.20% exposed pyrite) prepared from the surrounding rock or tailings and natural river sand is predicted to exceed the failure limit (0.040% expansion threshold) within 1.28–2.81 years. In comparison, concrete incorporating fine aggregates (containing ≥4.10% exposed pyrite) prepared from the surrounding rock or tailings and limestone is predicted to exceed the failure limit within 0.96 to 1.47 years.

According to the constant y_0_ (<0.04%), when the surface-exposed pyrite content in coarse aggregates from the surrounding rock or tailings is 2% or less, C50 concrete prepared with such coarse aggregates and natural river sand will not fail due to pyrite oxidation. The threshold limit for fine aggregates from the surrounding rock or tailings is set at 3% or less. Notably, sulfate attack induced by pyrite oxidation can trigger expansion or cracking, compromising structural integrity. Based on the mix design of concrete in Table 4, the calculated limit for surface-exposed pyrite in C50 concrete is 21.45 kg/m^3^. Restricting this content to below 20 kg/m^3^ can effectively mitigate these adverse effects. As ettringite is a key factor in expansion-induced cracking, the safety threshold for pyrite content may change depending on the C_3_A content in the cement.

## 4. Conclusions

While the detrimental effects of pyrite-containing aggregates on concrete are widely recognized, the underlying processes remain complex and poorly understood. Through a novel approach, we prepared concrete utilizing standard engineering mix designs with coarse or fine aggregates derived from the surrounding rock or tailings. The findings elucidate the mechanisms triggering pyrite-induced degradation, establish a robust concrete failure prediction model, and propose a safe threshold for pyrite content in aggregates.

(1) The effect of curing temperature on concrete expansion caused by pyrite oxidation follows the Arrhenius equation. The activation energy for concrete expansion ranges from 8.28 to 9.47 kJ/mol.
(2) Pyrite exposed on the surface of concrete aggregates undergoes inward oxidation, resulting in the formation of the oxidation product goethite. Conversely, pyrite that is entirely encapsulated within other minerals in the aggregates remains stable and generally does not oxidize.
(3) An increase in curing temperature accelerates oxygen migration and promotes pyrite oxidation, leading to the formation of goethite and ettringite, which subsequently induces expansion and deterioration of the concrete.
(4) When the content of surface-exposed pyrite introduced from aggregates is 20 kg/m^3^ or less, the concrete will not be damaged by pyrite oxidation. If this limit is exceeded, sulfate erosion caused by pyrite oxidation may lead to expansion or even cracking, thereby influencing the strength of concrete.

## Figures and Tables

**Table 1:** Aggregates were selected for concrete specimens cured at 70 °C.

No.	Coarse Aggregate	Fine Aggregate	Curing Temperature/°C
1	GRR4	Natural River Sand	70
2	HRR1		
3	HTHR1		
4	HRR8		
5	GRR7		
6	GTR1		
7	HRR6		
8	GTR3		
9	Crushed Limestone	GTS3	70
10		HRS1	
11		HRS2	
12		HTHS3	
13		HTS1	
14		GRS8	
15		HRS6	

**Table 2:** Aggregates were selected for concrete specimens cured at different temperatures.

No.	Coarse Aggregate	Fine Aggregate	Curing Temperature/°C
1	GRR2	Natural River Sand	20, 30, 40, 50, 60, 70
2	Crushed Limestone	HTLS3	
3	Crushed Limestone	Natural River Wand	

**Table 3:** Chemical composition of cement and admixtures.

No.	Material	Chemical Compositions/%								
		SiO_2_	Al_2_O_3_	Fe_2_O_3_	CaO	MgO	K_2_O	Na_2_O	SO_3_	Loss
1	Cement	20.54	4.89	3.03	63.22	3.51	0.56	0.21	2.45	1.60
2	Fly Ash	50.53	31.65	4.48	4.07	0.92	1.26	0.68	1.32	2.77
3	Mineral Powder	33.72	17.74	0.77	38.00	6.35	0.41	0.41	1.04	−0.72

**Table 4:** The mix design of C50 concrete.

Strength of Concrete	Mix Design/(kg/m^3^)							W/B Ratio
	Cement	Slag	Fly Ash	Sand	Rock	Superplasticizer	Water	
C50	330	60	70	715	1042	2.3	163	0.35

**Table 5:** The expansion rate constant and correlation coefficient of concrete.

No.	Strength of Concrete	Temperature /°C	GRR2		HTLS3	
			Expansion Rate Constant k/(%/d)	Correlation Coefficient	Expansion Rate Constant k/(%/d)	Correlation Coefficient
1	C50	20	−0.11 × 10^−4^	0.59430	0.04 × 10^−5^	0.05838
2		30	0.57 × 10^−4^	0.99553	0.18 × 10^−4^	0.98820
3		40	0.89 × 10^−4^	0.98154	1.00 × 10^−4^	0.98870
4		50	1.09 × 10^−4^	0.98897	1.19 × 10^−4^	0.98732
5		60	1.20 × 10^−4^	0.98635	1.25 × 10^−4^	0.97429
6		70	1.25 × 10^−4^	0.97690	1.34 × 10^−4^	0.97628

**Table 6:** Activation energy of concrete expansion.

No.	Aggregate	Strength of Concrete	Activation Energy/kJ·mol^−1^	Correlation Coefficient
1	GRR2	C50	9.47	0.94405
2	HTLS3		8.28	0.96811

**Table 7:** The pyrite content of samples.

No.	Coarse Aggregate	Fine Aggregate	Total Pyrite Content/%	Content of Exposed Pyrite/%	364-Day Expansion Rate/%
1	GRR4	Natural River sand	2.44	0.74	0.012
2	HTHR1		2.45	0.99	0.023
3	HRR1		3.20	1.74	0.025
4	GRR7		4.77	1.85	0.028
5	GTR1		4.88	3.34	0.034
6	HRR6		5.61	4.23	0.040
7	HRR8		5.62	3.20	0.045
8	GTR3		7.11	3.78	0.048
9	Limestone	HRS1	0.32	0.22	0.002
10		HRS2	2.48	1.17	0.022
11		GTS3	5.27	3.27	0.033
12		HTS1	6.01	5.55	0.041
14		GRS8	10.06	4.10	0.048
15		HRS6	12.93	6.13	0.055

**Table 8:** The fitting results of the C50 concrete expansion ratio (exceeding 0.04%) and curing time.

No.	Coarse Aggregate	Fine Aggregate	The Content of Surface-Exposed Pyrite/%	Fitting Function	Correlation Coefficient
1	HRR8	Natural River Sand	3.20	E = 0.043 − 0.056exp(−t/198.80)	0.99848
2	GTR1		3.34	E = 0.049 − 0.065exp(−t/183.97)	0.99796
3	GTR3		3.78	E = 0.058 − 0.073exp(−t/221.81)	0.99694
4	HRR6		4.23	E = 0.058 − 0.075exp(−t/186.27)	0.99767
5	Crushed Limestone	GRS8	4.10	E = 0.052 − 0.060exp(−t/203.75)	0.99761
6		HTS1	5.55	E = 0.060 − 0.080exp(−t/196.26)	0.99723
7		HRS6	6.13	E = 0.065 − 0.078exp(−t/187.35)	0.99832

**Table 9:** Time to reach 0.040% expansion for C50 concrete.

No.	Coarse Aggregate	Fine Aggregate	The Time at Which the Expansion Rate Reaches 0.040%/y	
			70 °C	20 °C
1	HRR8	Natural River Sand	1.59	2.81
2	GTR1		1.00	1.76
3	GTR3		0.86	1.51
4	HRR6		0.73	1.28
5	Crushed Limestone	GRS8	0.90	1.47
6		HTS1	0.75	1.22
7		HRS6	0.58	0.96

## Data Availability

The original contributions presented in this study are included in the article. Further inquiries can be directed to the corresponding authors.
